# Current advancement in the application of prime editing

**DOI:** 10.3389/fbioe.2023.1039315

**Published:** 2023-02-16

**Authors:** Zhangrao Huang, Gang Liu

**Affiliations:** ^1^ State Key Laboratory of Mycology, Institute of Microbiology, Chinese Academy of Sciences, Beijing, China; ^2^ University of Chinese Academy of Sciences, Beijing, China; ^3^ The Innovative Academy of Seed Design, Chinese Academy of Sciences, Beijing, China

**Keywords:** prime editing, editing strategies, application, optimization, CRISPR-Cas9

## Abstract

Prime editing (PE) is a precise genome manipulation technology based on the “search and replace” approach of the CRISPR-Cas9 system, while it does not require the exogenous donor DNA and the DNA double-strand breaks (DSBs). Comparing the base editing technology, the editing scope of prime editing has been widely expanded. Prime editing has been successfully applied in a variety of plant cells, animal cells and the model microorganism *Escherichia coli* so far, and it has shown a good application potential in breeding and genomic functional study of animals and plants, disease treatment, and modification of the microbial strains. In this paper, the basic strategies of prime editing are briefly described, and its research progress is summarized and prospected from the application of multiple species. In addition, a variety of optimization strategies for improving its efficiency and specificity of prime editing are outlined.

## 1 Introduction

The CRISPR-Cas9 system composed of the clustered regularly interspaced short palindromic repeats (CRISPR) and its associated protein (Cas) has been found to exist in more than 50% of bacteria and more than 90% of archaea, and participate in the adaptive/acquired immune response in prokaryotic cells to defend against the exogenous plasmids or phages (McGinn and Marraffini, 2019). Due to its simplicity, convenience, low cost and high efficiency editing, the genome manipulation technology based on the CRISPR-Cas system has been developed and widely used in different species including animals, plants and a variety of microorganisms.

In the genome editing system based on CRISPR-Cas, the designed RNA guides the Cas nuclease to target the genome-specific sequence and generate DNA double strand break (DSB) which triggers the cellular repair by the non-homologous end joining (NHEJ) and the homology-directed repair (HDR) (Miyaoka et al., 2016). In eukaryotes, NHEJ is not only the main repair pathway, but also an imprecise repair system. Because NHEJ could produce the random insertion or deletion (indel), it usually causes frame shift mutation, resulting in the corresponding gene knockout or blocking. HDR is a precise repair system, which requires the exogenous donor DNA as template for the precise replacement, deletion, insertion or point mutation (Chapman et al., 2012). However, the introduction of DSB by CRISPR-Cas usually leads to production of the complex indel by-products, translocation and chromosome fragmentation, as well as the activation of p53 (Haapaniemi et al., 2018). To overcome this major problem, a single-stranded DNA (ssDNA) as donor was rationally designed and applied in the CRISPR-Cas9 system (Richardson et al., 2016). After optimizing the orientation, polarity and length of the donor ssDNA, the HDR rate was increased to 60%. Further, the correctly targeted alleles were significantly improved in mice through *Easi*-CRISPR with a long single-stranded DNA donor (Quadros et al., 2017). Although optimization by different strategies could partially solve the above problem, this technology based on the standard CRISPR-Cas system still has some problems such as the low editing efficiency and inaccurate editing. Therefore, it is urgent to explore more accurate genome manipulation strategies.

As the molecular mechanism of CRISPR-Cas system is becoming better understood, the genome editing based on CRISPR-Cas has been continuously improved and optimized. Various genome manipulation technologies based on the CRISPR-Cas system were emerged, which significantly improved the efficiency and accuracy of genome editing and reduced the off-target editing. Based on the CRISPR-Cas system, the base editing systems (BEs) were developed through integrating dead Cas9 (dCas9) or Cas9 nickase (nCas9) with different nucleoside deaminases by David Liu’s group in Bode Institute of Harvard University (Rees and Liu, 2018). According to the action types of deaminase linked with dCas9 or nCas9, the BE systems are classified as the cytosine base editors (CBEs) and the adenine base editors (ABEs). The part of nCas9 with single strand cleavage activity or dCas9 without cleavage activity is used to identify the DNA target sequence and the cytosine nucleoside deaminase or the adenine nucleoside deaminase is used to perform base replacement. With the BE technology, four base substitutions (C to T, G to A, A to G, and T to C) were fulfilled successfully (Gaudelli et al., 2017), while there was no need to produce the DNA double strand breaks or provide the exogenous donor DNA as template (Komor et al., 2016). However, the BE technology can only manipulate in a narrow window and generally produce synonymous mutation and is limited by the targeting scope of Cas. In addition, the base editing product purity is dependent on the uracil N-glycosylase inhibitor (UGI) which activity is different in various species. Off-target editing is still a major problem to be solved in base editing.

**FIGURE 1:**
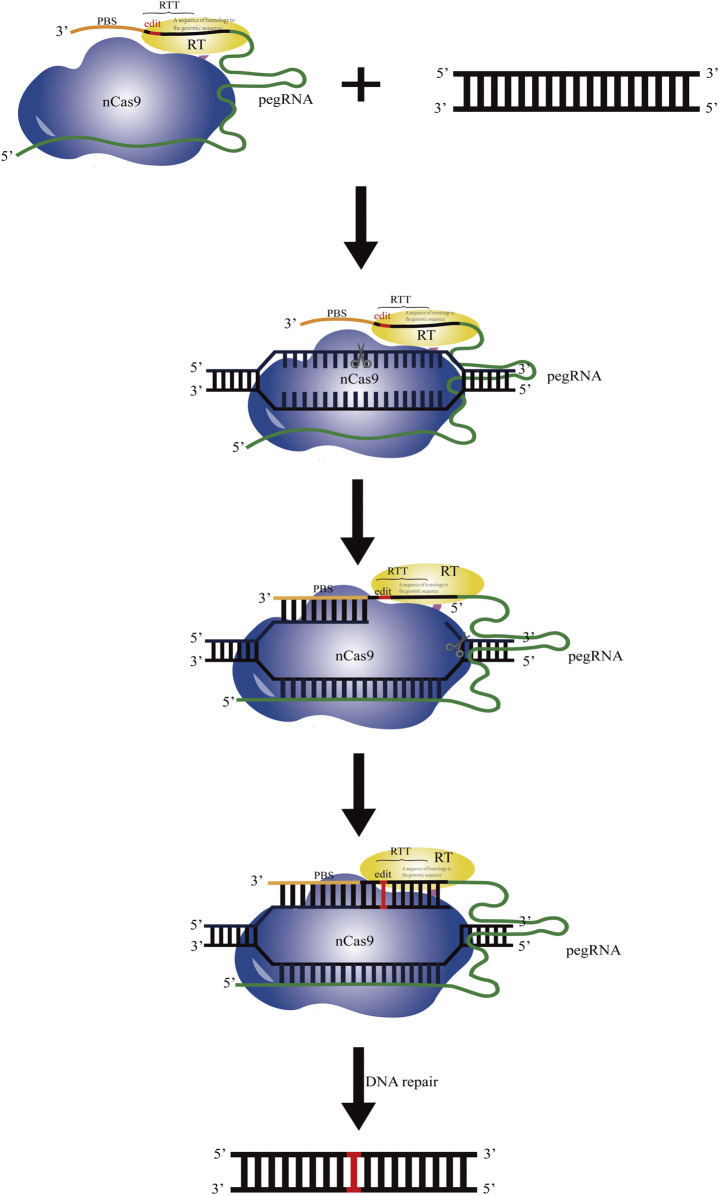
Schematic diagram of the operating principle based on the prime editing system. The PE system consists of the effecters formed by integrating nCas9 (H840A) (indicated in blue) and the moloney murine leukaemia virus reverse transcriptase (M-MLV-RT) (indicated in yellow) and the prime editing guide RNA (pegRNA). Under guidance of the sgRNA sequence in pegRNA, nCas9 cuts the DNA single strand. The primer binding sequence (PBS, indicated in brown) at the 3′end of pegRNA recognizes and pairs with the complementary sequence before the cutting site. Using the artificially designed template sequence downstream of PBS in pegRNA as the template, reverse transcription is performed by M-MLV-RT, and the target sequence is replaced.

In 2019, David Liu’s group developed a new technology named prime editing (PE), which significantly reduced the off-target editing and increased the scope of genome editing (Anzalone et al., 2019; Marzec et al., 2020). PE is a precise genome editing technology that relies on “search” and “replace” of the target sequences. The PE system consists of the effecter protein formed by integrating nCas9 (H840A) and the moloney murine leukaemia virus reverse transcriptase (M-MLV-RT), and the prime editing guide RNA (pegRNA) (Li and Huang, 2022). The pegRNA consists of an engineered standard sgRNA targeting the specific genome sites, an extension sequence including a primer binding site (PBS) at the 3′end of sgRNA, and the reverse transcription template (RTT) with the desired editing sequence. In the PE system, nCas9 with the single strand cleavage activity is fused with M-MLV-RT and targets to the specific sites mediated by pegRNA. Then a single strand on the target site is cut by the effecter protein and a nick is generated, and the reverse transcription starts dependent on the RTT in pegRNA. Then, the edited DNA sequence is integrated into the complementary chain through the DNA repair mechanism, and the edited DNA is further copied into another chain to produce a stable edited DNA sequence (Figure 1). All 12 possible base to base conversions including four kinds of transition point mutations, eight kinds of transversion point mutations, insertion (up to 44 bp), deletion (up to 80 bp), and combined editing of the above four methods, can be fulfilled using the PE technology. It has the advantages that no DSB or exogenous donor DNA is required. It has accuracy and flexibility in editing and less indel by-products are produced. Meanwhile, it has a large gene editing range and strong editing ability.

In order to increase editing efficiency of the PE system, David Liu’s group successively improved the editing systems in several advanced versions such as PE1, PE2, PE3, and PE3b (Anzalone et al., 2019). In the first developed prime editing technology (PE1), M-MLV-RT was fused into the C-terminal of nCas9 to generate the effecter protein. Although PE1 could accurately edit the designed sequence of genome, it had a low editing efficiency in mammalian cells. In the PE2 system, six types of point mutations (H9Y, D200N, T306K, W313F, T330P, and L603W) in M-MLV-RT of the effecter protein were introduced. The affinity and stability of RNA-DNA substrate were significantly improved in the optimized M-MLV-RT. Based on PE2, the PE3 system was further developed. By introducing a nicking sgRNA, a nick was produced in the non-edited strand and the second cutting was occurred at different distances. Through stimulating replacement of the non-edited strand, the editing efficiency was further improved in PE3. Although the editing efficiency of PE3 was higher than that of PE2, but the risk of producing indels by-products also increased due to introduction of nick in the non-edited strand. By increasing the specificity of sgRNA for the edited sequence, the PE3b system further reduced the indel by-products caused by introduction of the nicking sgRNA and improved the accuracy of genome editing.

Recently, several comprehensive reviews on progress of the PE strategies and applications have been published (Bharucha et al., 2022; Chen & Liu, 2022; Doman et al., 2022; Lu et al., 2022; Raguram et al., 2022). The PE strategies for genome manipulation and their limitations are summarized. In this review, we mainly focus on the application and optimization of the PE strategies in different species.

## 2 Application of the PE system

Like other genome editing approaches based on the CRISPR-Cas system, PE has been quickly applied in engineering of the living cells as soon as it emerged (Table 1). PE was first applied in the mammalian cells and it was found in the original PE system could correct up to 89% of known gene variants related to human diseases (Anzalone et al., 2019). In addition to its application in the mammalian cells, the PE derivative technologies have been rapidly developed and applied in plants, and it shows great potential in promoting plant genome editing which is impeded by the low homologous recombination frequency and the limited donor transmission (Hassan et al., 2021). Microorganisms are not only used as tools for studying the life science, but also closely related to human health, industrial and agricultural productions. Recently, PE was also applied in the model microorganism *Escherichia coli* (Tong et al., 2021). With the sustainable development of PE system, it will be widely used in various fields of life science.

**TABLE 1:** Application of PE in different species.

Species	Application		Reference
Plant cells	Monocotyledon	Rice and wheat	Lin et al. (2020), Jin et al. (2021), Xu et al. (2021), Lin et al. (2021)
		maize	Jiang et al. (2020)
	Dicotyledon	*Arabidopsis thaliana*	Wang et al. (2021)
		Tomato	Lu et al. (2021)
		Potato	Perroud et al. (2022)
Animals	Non-mammalian animal models		Bosch et al. (2021), Petri et al. (2022)
	Mice		Liu et al. (2020), Billon et al. (2020), Gao et al. (2021), Liu et al. (2021a)
	Rabbit embryos		Qian et al. (2021)
	Human cells		Anzalone et al. (2022), Bharucha et al. (2022), Eggenschwiler et al. (2021), Kim et al. (2021), Morris et al. (2021), Petri et al. (2022), Schene et al. (2020), Sürün et al. (2020), Wolff et al. (2021), Zhuang et al. (2022)
Microorganism	*Escherichia coli*		Tong et al. (2021)

### 2.1 Application of the PE system in plants

Due to the low efficiency of homologous directed repair (HDR) and the obstacles in providing sufficient donor repair templates (DRT) in the plant cells, accurate genome editing through HDR is challenging. Since PE does not require DNA double strand breaks (DSB) or DRT to fulfill accurate editing of the genome target sequence, it can be used to carry out the genome editing in plant cells and provide better technical support for crop breeding improvement.

#### 2.1.1 Application of PE in genetic breeding of rice and wheat

As main food crops, the stable and high yield of rice and wheat is very important for food security in the world. The studies on improving the yield and enhancing the resistance of rice and wheat against the plant pathogens through genetic breeding have attracted much attention. To achieve this goal, a PE derivative system called PPE, was constructed and successfully applied in the genome editing of rice and wheat respectively (Lin et al., 2020). The number and the location of mismatches in the PBS and spacer of the pegRNA influenced the editing frequencies and off-target edits had quite low frequencies (0.00%–0.23%) in rice. An improved PE system realized versatile nucleotides substitution in three genes (*OsALS*, *OsACC*, and *OsDEP1*) in rice by using U3 and U6a promoters to control the pegRNA and sgRNA, respectively (Xu et al., 2020). In addition, a systematic study was performed to detect the editing specificity of PPEs at the genome-wide level (Jin et al., 2021). A prime editing library mediated saturation mutagenesis (PLSM) method was developed to increase the diversity of amino acid substitutions for plant screening and applied to evolve herbicide-tolerance genes in rice (Xu et al., 2021).

#### 2.1.2 Application of PE in genetic breeding of maize

In rice, regenerated prime edited frequencies reached to 21.8%, but most of the mutants were found to be the chimeric mutations. By optimizing the expression of pegRNA, the editing efficiency was improved in the acetolactate synthase (ALS) coding gene of the maize cells (Jiang et al., 2020). To produce the herbicide resistant maize lines containing the P165S mutation or the W542L/S621I double mutation in *ZmALS1* and *ZmALS*2, a PE system containing two pegRNA variants was constructed for targeting *ZmALS1* and *ZmALS*2 of maize to cause the double mutations. The results showed that 53.2% and 6.5% of the transgenic lines carried S621I and W542L mutations in *ZmALS1* and *ZmALS*2, respectively. For these two *ALS* genes, 4.8% of the transgenic lines contained the homozygous S621I mutation and the W542L/S621I double mutation, respectively. Compared with rice, a higher editing efficiency was achieved in maize. To further improve the editing efficiency, it is necessary to optimize the design of pegRNA and increase the expression of pegRNA.

#### 2.1.3 Application of PE in genetic breeding of dicotyledons

Application of the genome editing technology based on the CRISPR-Cas9 system can accelerate the domestication of the dicotyledonous plants (such as tomato) and improve their yield and their resistance to biotic or abiotic stresses. By using the modified prime editor called pCXPE03, the precise genome modifications were achieved in tomatoes (Lu et al., 2021). The *RPS5A* promoter of tomato was used to express the plant codon-optimized MMLV (pMMLV), while the pegRNA and sgRNA were driven by the U6 promoter of *Arabidopsis* in pCXPE03. By testing the editing efficiency of pCXPE03 in three tomato genes (*GAI*, *ALS2*, and *PDS1*), it was further verified that the optimized pCXPE03 could be used to edit the endogenous genes in tomato cells, meanwhile 0.5%–4.9% of off-target by-products were generated.

These studies raise the possibility that PE has wide application prospects in plants. However, the relatively low editing efficiency of PE in plants limits its application in plant biotechnology and agricultural. Thus, it is urgently needed to develop an efficient genome editing system. It indicates that further optimizing the PE system is required for editing the plants including effecter structural evolution, pegRNA optimization, and system redesign.

### 2.2 Application of PE in genome editing of animals

#### 2.2.1 Application of PE in non-mammalian animal models

Since no require the exogenous donor DNA and double strand DNA breaks, PE was applied in the non-mammalian animal model insect *D. melanogaster*. To test feasibility of the PE system, the PE elements were expressed in the *Drosophila* derived S2R^+^ cells (Bosch et al., 2021). It was found that the editing efficiency of PE was the highest in the Act > PE2 cell line. Using PE, the stop codon was accurately introduced into the three marker genes (ebony, white, and forked genes), and the editing efficiency reached to 35.2, 11.6, and 21.9%, respectively. To use the PE system in the germline to create and reproduce the edited *D. melanogaster* population, the PE2 transgenic *D. melanogaster* was prepared in the form of a combination of a single transgene (nos-PE2 or nos-Gal4) and UAS-PE2 (NOS > Pe2) under the control of the germ cell specific NANO gene promoter. The results showed that the effective germ line could be accurately edited in the ebony gene of *D. melanogaster* and it was transmitted to the off-spring with 36% efficiency. *D. melanogaster* is the first non-mammal model to test this method and this study shows the potential application value of the PE system in other non-mammal animals.

As an animal model used for studying the human diseases, zebrafish is one of the excellent vertebrate materials and has been widely used. Recently, a zebrafish model was constructed for human disease study by using the purified ribonucleoprotein complex (PE-RNP) system (Petri et al., 2022). The pegRNAs with the different lengths of RTT and PBS were used to target the *tyr1* and *tyr2* sites in the zebrafish tyrosinase gene. Based on the editing efficiency of the PE-RNP system in the zebrafish embryonic cells, the RTT of 13–15 nt was more suitable for editing the zebrafish tyrosinase gene. However, off-targeted insertion, deletion and pegRNA chimerism were observed when up to 30% somatic mutations were introduced into the zebrafish embryos. It indicated that the purified PE-RNP complex could induce somatic and germline genetic mutations in zebrafish. The successful transmission in zebrafish mutation induced by the PE system also demonstrates that PE has potential application value in constructing other vertebrate disease models.

#### 2.2.2 Application of PE in mammalian models

By using the PE technology, the base conversion of target sequence in mice was successfully carried out (Billon et al., 2020; Liu et al., 2020; Gao et al., 2021; Liu et al., 2021a). At the same time, off-target editing was found. To induce the point mutation in the X-linked androgen receptor encoding gene *Ar* and the homeobox protein Hoxd13 encoding gene in the mouse N2a cells by using the PE3 system, the pegRNAs and sgRNAs were designed and the editing conditions were optimized. After the mRNA encoding pCMV-PE2 and different pegRNA with the corresponding nicking sgRNA were co-injected into the single-cell embryos of mouse, the base conversion in *Hoxd13* was observed in 44% and 75% of blastocysts, respectively. It was also found that there was off-targeted mutation with frequency of 1.1%–18.5%, suggesting that the fidelity of the PE3 system in mouse embryos was low. Therefore, it was necessary to screen the suitable pegRNAs *in vitro* before using the PE system for genome editing *in vivo*. Then embryonic cells containing pCMV-PE2 and pegRNAs for *Hoxd13* editing were implanted into the surrogate female mice. The results showed that the frequency of insertion and deletion dependent on PE *in vivo* was low, indicating that zygotic injection induced the formation of somatic chimera and increased the complexity of editing. To further detect the off-targeted mutations in the PE edited mice, Cas-OFFinder11 was used and 16 off-targeted mutation sites were identified, respectively. Even so, the above results showed that the PE3 mediated base conversion was highly specific *in vivo* compared with the base editing system. Thus, PE could be a good selection in genome editing of mice.

Since its emerging, PE has been applied in human cells (Anzalone et al., 2019). In HEK293, the transversion point mutations were successfully achieved with efficiencies of 0.7%–5.5% by using PE. Trough optimizing the RT structure, PE2 was generated and it demonstrated a good ability in transversion and insertion editing. PE3 with a sgRNA for nicking the non-edited strand further improved the editing efficiency. Further, the multiple functions of the PE3 system in HEK293T was verified and found that base conversion was successfully generated in six target sites (Liu et al., 2020). It recently reported that the preliminary editing with the PE-RNP system introduced the targeted mutations at a frequency of 21% and 7.5% in HEK293T and the primary human T cells, respectively (Petri et al., 2022). In addition, the editing efficiency of PEs (PE2, PE3, and PE3b) in 15 loci of the HEK293FT cells was determined (Qian et al., 2021). Of them, base insertion occurred at 5 loci (frequency 4%–22%), base substitution occurred at 8 loci (frequency 4%–36%), and base deletion occurred at 2 loci (frequency 7% and 12%, respectively). These results indicated that the PE system could effectively introduce the base insertion, substitution and deletion in the target sites in human cells.

Tay-Sachs disease (TSD) is an autosomal recessive disease in human. It is caused by *HEXA* (encoding β-aminohexosidase A) disorders and characterized by abnormal sphingolipid metabolism. In order to study the treatment of the disease, a new rabbit TSD model was constructed by using the PE system (Qian et al., 2021). Many genetic diseases are caused by insertion or deletion of DNA fragments in the genome. To solve this problem, a PE-Cas9-based deletion and repair (PEDAR) technology was developed and applied in HEK293T cells (Jiang et al., 2022a). It demonstrated that PEDAR was better than other genome editing methods in editing the report genes and the endogenous genes, and it can effectively produces large and accurate genome changes. Comparing PE, PEDAR could delete more than 10 kb DNA fragment and insert up to 60 bp DNA fragment. In addition, PEDAR was further applied to revise the mutation which causes tyrosinemia in hepatocytes, demonstrating that PEDAR could be used for repairing pathogenic mutations in genetic diseases.

The PE system is mainly applied in potentially correcting gene mutations that cause human diseases and producing animal models. Since about 90% of human pathogenic gene variants are single base mutations or insertion and deletion of less than 12 base pairs, which is a type of DNA change within the capability of the basic editing system. Although these studies illustrate the potential of preliminary editing in disease treatment, transmitting it to other organs, such as the heart, is still the main challenge for application of the therapeutic preliminary editing. Moreover, the low efficiency of delivery and transmission of somatic chimeras is also the difficulty in producing animal models. Therefore, innovative delivery technology and the improvement of PE system are crucial to maximize the efficiency of primary animal editing.

### 2.3 Application of PE in microorganism

Microorganisms, including bacteria, archaea, and the eukaryotic microorganisms such as yeast and filamentous fungi, widely exist in nature. Besides some pathogenic microorganisms that can cause diseases of human, animals and plants, most microorganisms are of benefit to the industrial and agricultural productions and human health. Some microorganisms are also good materials for the basic life science research. *Streptomyces*, as the filamentous bacterium, is the main source of most anti-infective drugs in clinic. More and more microbial genome sequences revealed that *Streptomyces* contains many recessive gene clusters and is still an important source of active metabolites (Liu et al., 2013). Eukaryotic microorganisms, including yeast and filamentous fungi, can produce a variety of proteins and metabolites, so they are important industrial microorganisms.

The CRISPR-Cas9 system not only comes from microorganisms, but also has been used in microbial genome editing since its emergence. In 2013, the CRISPR-Cas9 system was introduced in *Saccharomyces cerevisiae* and greatly improved the targeted editing ability of the genome (DiCarlo et al., 2013). In the same year, the genome of *Streptococcus pneumoniae* and *E. coli* were successfully edited by using the CRISPR-Cas9 system (Jiang et al., 2013). Subsequently, the CRISPR-Cas9 system has been applied in a variety of filamentous fungi (Song et al., 2019; Rozhkova and Kislitsin, 2021). In 2015, the CRISPR-Cas system was applied in *Aspergillus nidulans* (Nødvig et al., 2015), following application in *Penicillium chrysogenum*, *Trichoderma reesei*, *Neurospora crassa*, *Acremonium chrysogenum* and so on (Liu et al., 2015; Matsu-Ura et al., 2015; Pohl et al., 2016; Chen and Chu, 2019; Chen et al., 2020). Recently, the base editing system fusing cytidine deaminase and Cas9 nickase was developed and applied in *A. niger* (Huang et al., 2019). However, the problems such as its natural narrow editing window and off-target still exist in filamentous fungal genome editing using the BE system.

**FIGURE 2:**
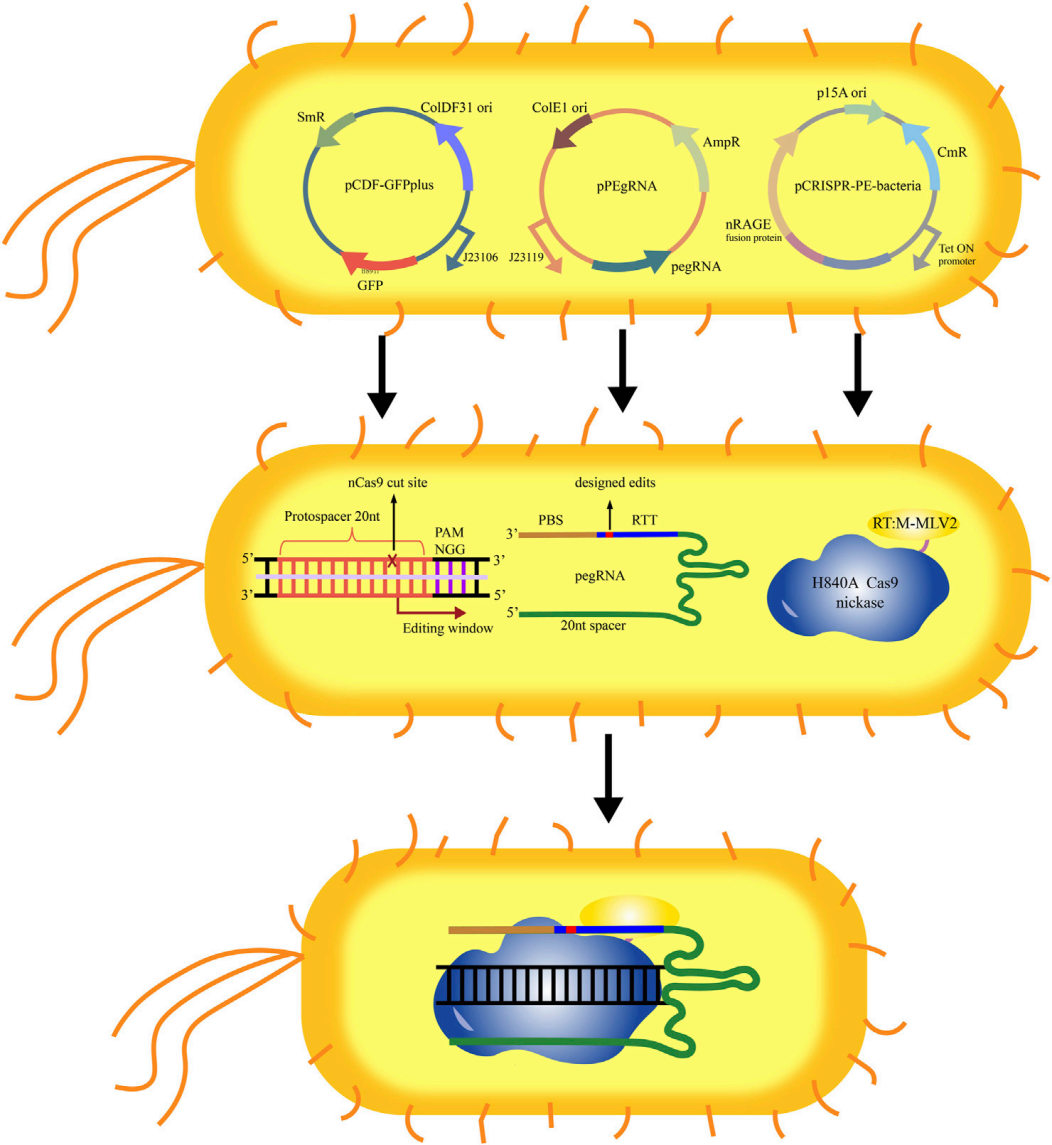
Schematic diagram of the operating principle of the PE system in *E. coli*. The plasmid pCDF-GFPplus was used for expression of *GFP,* which could be used as the editing target. The plasmid pPEgRNA was used for expression of pegRNA. The plasmid pCRISPR-PE was used for expression of nCas9-M-MLV2. After introduced the three plasmids (pCDF-GFPplus, pPEgRNA, and pCRISPR-PE) into *E. coli*, the nCas9-M-MLV2:PEgRNA complex binds to the targeted sequence guided by sgRNA. The PBS recognizes and hybridizes to the single stranded DNA, and starts the reverse transcription of new DNA containing the designed edits based on the RTT.

As a model microbial material, *E. coli* has been widely used in basic research and applications in agriculture and industry. Although many tools have been developed for the genetic manipulation of *E. coli*, the efficient and convenient genome editing tools are still insufficient. Due to lack of non-homologous end junction (NHEJ) system in most prokaryotes, the early genome editing based on the CRISPR-Cas9 system depends on the existence of the homologous DNA donors, which increases the difficulty in the *E. coli* genome editing. Recently, a genome editing kit for the prokaryotic microorganisms such as *E. coli* based on the PE system was developed (Tong et al., 2021). This genome editing kit included a three-plasmid system (pCDF-GFPplus, pPEgRNA, and pCRISPR-PE), in which pCDF-GFPplus was used as a report plasmid to detect the editing efficiency of PE in *E. coli* (Figure 2). Further the editing conditions including induction conditions, the optimal length of PBS and RTT were optimized, and the ability of the optimized PE system was evaluated in editing the genome of *E. coli* through detecting the mutation of lactose metabolism pathway and D-galactose metabolism pathway, and the targeted mutation and potential off-targeted mutation were also evaluated by using single nucleotide polymorphism (SNP) analysis method. Under the optimal conditions, the single base deletion efficiency was as high as 40%. In addition, the DNA sequences up to 97 bases were successfully deleted in *E. coli*, and the sequences up to 33 bases were inserted by using this genome editing kit. However, the editing efficiency of PE in *E. coli* was still very low. This work is only a preliminary attempt of PE system in prokaryotic microorganisms. By optimizing the conditions, it is expected to significantly improve the efficiency of genome editing. At the same time, it is also looked forward to the application of the PE system in more different kinds of microbial cells.

## 3 Optimization of the PE system

**FIGURE 3:**
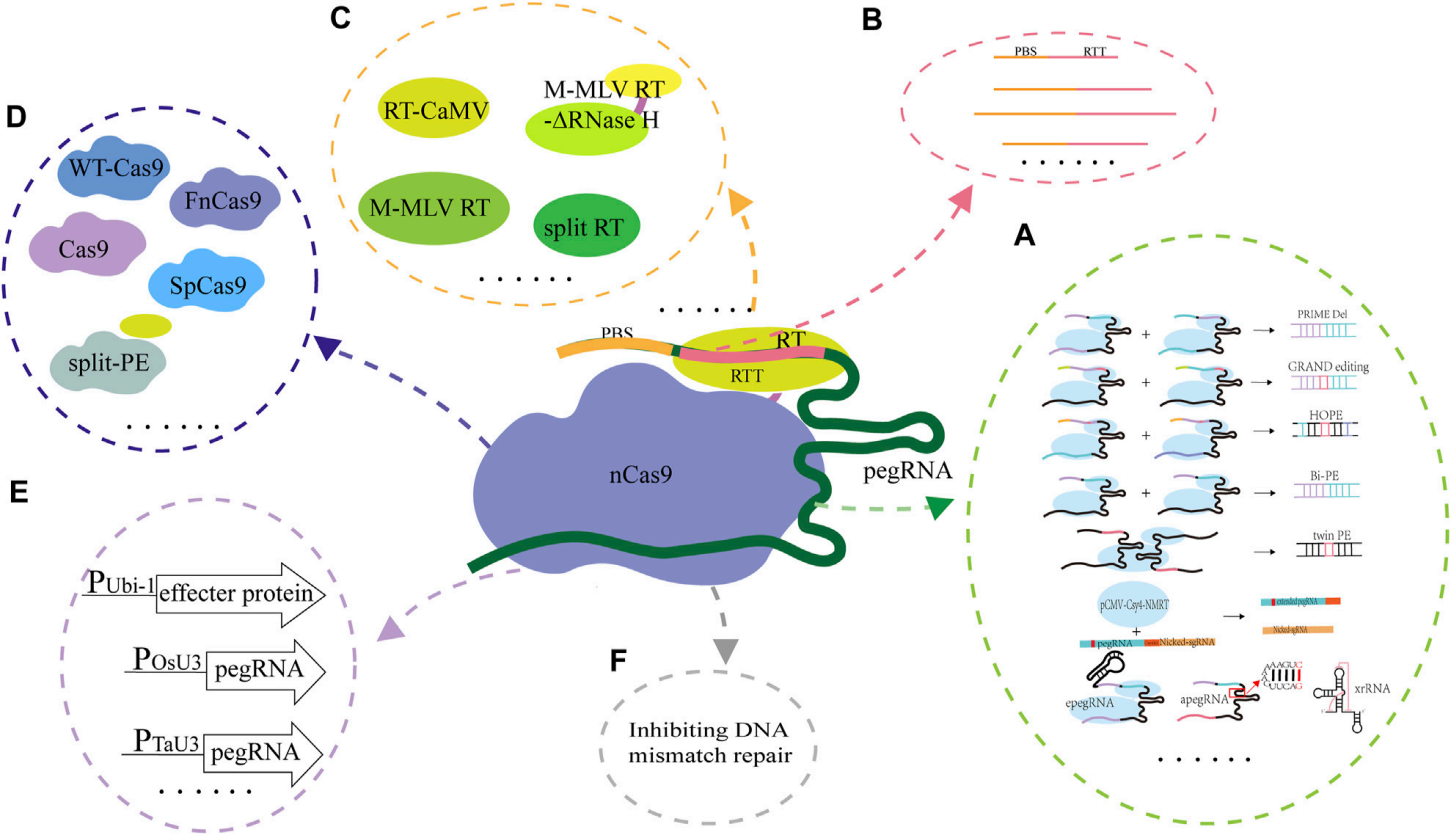
Strategies used for optimization of the PE system. **(A)**, Optimization of pegRNA. In PRIME-Del, a pair of pegRNAs for opposite DNA strands were used for accurately editing the targets; In GRAND, a pair of pegRNAs with different but complementary RTT and target DNA sequences were used; In HOPE, two pegRNAs which contain homologous 3′terminals and used for targeting DNA double strands were designed and used; In Bi-PE, a nick sgRNA near the pegRNA template sequence was used; In twinPE, a prime editor protein and two pegRNAs were used; In ePE, the pegRNA skeleton was optimized through fusing the nicking sgRNA and pegRNA in one transcriptional unit; In epegRNA, the pegRNA (epegRNAs) was engineered based on integration of the structured RNA motifs into the 3′end of pegRNAs; **(B)**, Changing the lengths of PBS and RTT. The ratio of targeted editing to off-target editing was significantly affected by the length of RT template, but did not change with the length of PBS or the location of nicking sgRNA; **(C)**, Optimization of RT. In the ePPE system, the RNase H domain in M-MLV RT was deleted and a virus nucleocapsid protein (NC) was added; In the PPE system, the engineered M-MLV-RT was replaced by CaMV-RT from cauliflower mosaic virus and reverse transcriptase derived RT (RT retron) from *E. coli* BL21; In Spilt-PE, the fusion effecter protein was split into two parts including split nSpCas9 and MMLV-RT; **(D)**, Optimization of Cas9 for expanding its editing scope. The PE2 variants (PE2-vqr, PE2-vrqr, PE2-vrer, PE2-ng, PE2-spg, and PE2-spry) could recognize the different PAM sequences; **(E)**, Optimization of the promoters for expressing the effecter protein gene and pegRNA. In PPE, the ubiquitin-1 gene (*Ubi-1*) promoter from maize was used to drive the expression of the effecter protein coding gene after its codon was optimized according to the codon preference of rice, the *osu3* promoter from rice (or the *TaU6* promoter from wheat) and the *TaU3* promoter from wheat were used to drive the transcription of pegRNA and the nicking sgRNA; **(F)**, Optimization of the PE system through inhibiting the DNA mismatch repair. The PE4 and PE5 systems were constructed through transient expressing the DNA mismatch inhibitor protein MLH1dn coding gene; PEmax was constructed by changing RT codon, mutating SpCas9 and optimizing the NLS sequence. The ePE3max system consists of PEmax protein, an epegRNA with evopreQ1 and a nicking sgRNA, and the ePE5max system consists of ePE3max and a dominant negative OsMLH1 variant that inhibits MMR.

Although the PE system has been applied and evaluated in animals, plants and the model microorganism *E. coli*, its editing efficiency and accuracy are unsatisfactory. The effecter protein formed by fusing nCas9 and the reverse transcriptase and pegRNA are the core components of the PE system, and they are also the key factors for determining the successful application and the editing efficiency of the PE system. Since emergence of the PE system, optimizing and evaluating the design of effecter protein and pegRNA are ongoing (Figure 3). The studies on the collaborative optimization and adaptation between pegRNAs and the effecter proteins have been carried out continuously (Table 2).

**TABLE 2:** Strategies used for optimization of the PE system.

Strategies	Description	Results	References
Optimization of pegRNA			
PRIME-Del	The technology is used for accurately editing the targets based on a pair of pegRNAs for opposite DNA strands	Targeted deletion of sequences up to 10 KB with higher accuracy (1%–30%).	Choi et al. (2022)
GRAND	The technology is based on a pair of pegRNAs with different but complementary RTT and target DNA sequences	Its editing efficiency was 63.0% in 150 bp inserts but it could introduce a small amount of by-products, and its editing efficiency was reduced to 28.4% in 250 bp insertions	Wang et al. (2022)
Bi-PE	The Bi-PE system contains nick sgRNA near the pegRNA template sequence	The Bi-PE strategy can increase efficiency by 16 times and the editing accuracy by 60 times. The system can delete large DNA (100–100 bp) and can introduce small fragments of 10–100 bp into the deletion sites	Tao et al. (2022b)
HOPE	Two pegRNAs which contain homologous 3′terminals and used for targeting DNA double strands are designed in this system	HOPE provides a new choice of guided editing with a high balance between efficiency and accuracy	Zhuang et al. (2022)
twinPE	The system uses a prime editor protein and two pegRNAs	The length of genes that can be edited by twinPE has expanded to thousands of base pairs which are equivalent to the length of a complete gene	Anzalone et al. (2022)
ePE	The technology is based on the optimization of the pegRNA skeleton through deletion and replacement	Compared with the standard PE, ePE improves the editing efficiency of point mutation by an average of 1.9 times	Liu Y et al. (2021b)
epegRNAs	The engineered pegRNA (epegRNAs) based on integration of the structured RNA motifs into the 3′end of pegRNAs	The optimized PE system can increase the editing efficiency in HeLa, U2OS, K562 cells and primary human fibroblasts by 3–4 times without increasing the off-targeted editing activity.	Nelson et al. (2022)
xrPE	The xrPE platform is developed by adding an xrRNA to the 3′extension region of pegRNAs	The average enhancement in base transformation, small deletion and small insertion of pantarget was 3.1, 4.5, and 2.5 times, and xrPE has comparable edits/index ratios, and minimum deviation target edits	Zhang et al. (2022)
TM of PBS	Optimization of PBS and RT template length	When the TM of PBS was at about 30°C, the activity of PE was 1.5–4.3 times higher than that of the PBS with other TM	Lin et al. (2021)
Length of RT template		The ratio of targeted editing to off-targeted editing was significantly affected by the length of RT template, but did not change with the length of PBS or the location of nicking sgRNA	Lin et al. (2020)
apegRNA and spegRNA	The apegRNA is developed by improving the pegRNA secondary structure and the spegRNA is developed by introducing same-sense mutations (SSM) at proper positions	The frequency of indel increased, but the frequency of unexpected indel and the part of incomplete products and by-products were not significantly affected	Li et al. (2022).
Optimization of the effecter proteins			
PE2 variants	PE2 variants (PE2-vqr, PE2-vrqr, PE2-vrer, PE2-ng, PE2-spg, and PE2-spry) recognize the different PAM sequences	More than 50 types of PE2 variants were successfully generated in HEK293T cells, and their editing activity was as high as 51.7%. In addition, BRAF V600E mutation was successfully introduced, which could not be induced by traditional PE system	Kweon et al. (2021)
ePPE	The ePPE system is constructed by deleting the RNase H domain in M-MLV RT and adding virus nucleocapsid protein (NC)	The synergistic effect of the two modifications increased the efficiency of base substitution, deletion and insertion at different endogenous sites by an average of 5.8 times, while no significant increase in by-products or partial off-targeted editing was observed	Zong et al. (2022).
WT-PE	The WT-PE system is designed by fusing reverse transcriptase (RT) and nuclease wild-type Cas9	WT-PE has realized the efficient and multifunctional large-scale genome editing, including large-scale deletion of up to 16.8 Mbp and chromosome translocation. This system could generate bi-directional primer editing	Tao et al. (2022a)
FnCas9	A technique developed by connecting reverse transcriptase to the new *Francisella novicida* Cas9 (FnCas9)	The editable region of prime editing could be extended by different nicking properties of CRISPR-Cas orthologs and engineering the PAM recognition domain within the Cas9 nickase. It also expands the region recognized as RTT after PBS sequence for prime editing	Oh et al. (2022).
PepSEq	A technique developed by fusing peptides derived from DNA repair proteins to the N-terminal of PE2	Through peptide fusion, PepSEq significantly improved the prime editing efficiency	Velimirovic et al. (2022)
Split-PE	Fusion proteins are split into two parts: split nSpCas9 and MMLV-RT	This PE system can be tested faster without optimizing the length or relative position of the linker with the fusion protein. In addition, off-target effects of split PE2 is similar to that of PE2	Grunewald et al. (2022)
Collaborative optimization of PE with multiple strategies			
PEDAR	PEDAR is based on the Cas9 nuclease (but not nCas9) combined with the reverse transcriptase and a pair of pegRNA	PEDAR can introduce more than 10 kb target deletion and up to 60 bp insertion into cells. In the tyrosinemia mouse model, PEDAR removed the 1.38-kb pathogenic insertion in the *Fah* gene and accurately repaired the missing connection to restore the expression of the *Fah* gene in the liver	Jiang T et al. (2022a).
The optimized PE	In the optimized PE system, RT is fused at the N-terminal of nCas9 and multiple nucleotide substitutions are introduced into the reverse transcriptase template	The optimized PE system was applied in the transgenic rice plants (24.3%), the maize protoplasts (6.2%) and the human cells (12.5%), which was 2–3 times higher than PE3	Xu et al. (2022)
SPE	The pegRNAs are split into sgRNA and petRNA, replace M-MLV RT with two compact codon optimized bacterial RT, use a dual AAV vector strategy	The efficiency in installing accurate editing is similar to that of PE3 (30%), while indel by-products are not increased, and AAV delivery is simpler	Liu et al. (2022)
Optimization strategy based on inhibiting DNA mismatch repair			
PE4, PE5 and PEmax	The PE4 and PE5 systems are constructed through transient expressing the DNA mismatch inhibitor protein MLH1dn coding gene. PEmax is constructed by changing RT codon, mutating SpCas9 and optimizing the NLS sequence	The editing efficiencies of PE4 and PE5 systems are 7.7 times and 2.0 times higher than that of PE2 and PE3 respectively, and the editing ratio is 3.4 times higher	Chen et al. (2021)
Comprehensive optimization method including three strategies			
ePE3max and ePE5max	ePE3max consists of PEmax protein, an epegRNA with evopreQ1 and a nicking sgRNA, and ePE5max consists of the ePE3max system and a dominant negative OsMLH1 variant that inhibits MMR	The prime editing efficiency of rice is greatly increased by improving PEmax structure and epegRNA. In addition, the by-products derived from pegRNA scaffold can be eliminated by using the termination rule of designed pegRNA	Jiang et al. (2022)

### 3.1 Optimization of PE based on pegRNA

In the PE system, pegRNA and its 3′extension region are the key components which are used to guide the effecter protein to target the genomic site and fulfill the accurate genome editing. Comparing sgRNA, pegRNA is composed of two sequences at its 3′end. One of the sequences contains PBS, complements with the 3′end of the cleaved strand and is involved in starting the reverse transcription process. Another sequence is used as RTT and carries the sequences in which the point mutation, insertion or deletion is introduced (Figure 1). Modification of pegRNA is the most direct optimization strategy for enhancing the editing ability of PE system.

#### 3.1.1 Optimization by using double pegRNAs

Targeted deletion of the genomic DNA sequence based on the CRISPR-Cas9/double sgRNA system needs to generate DSB and connect breakpoints through NHEJ which generally causes the problems such as low efficiency and insufficient accuracy. To solve this problem, a targeted deletion genomic DNA sequence technology (PRIME Del) was established based on the PE system with double pegRNAs (Choi et al., 2022). This technology could accurately edit the target sequence by introducing two pegRNAs for two complementary DNA strands. Experiments showed that PRIME Del was more accuracy and flexibility in targeted deletion of the genomic sequence, targeted labeling the marker and the possible genomic rearrangement. Compared with the CRISPR-Cas9/double sgRNAs system, PRIME Del could delete up to 10 kb sequence with higher accuracy, and the editing efficiency reached to 1%–30%. PRIME Del was also used for combining the target genomic DNA sequence deletion with the short sequence insertion. In this study, it was demonstrated that the length of RTT had a significant impact on the editing efficiency. In addition, it was found that prolonging or improving the expression of PE elements greatly improved the targeted deletion efficiency of the genomic DNA without affecting the accuracy. A GRAND editing system was also developed by using a pair of pegRNAs (Wang et al., 2022). Among them, the RTT sequence located in pegRNA was not necessary to be homologous with the target DNA sequence, but the RTT sequences in the two pegRNAs are partially complementary. Due to the insertion of the target DNA sequence and the deletion of the sequence between the two gaps, GRAND could avoid the competition between the original sequence and the new transcriptional sequence in the genome. Using this system, the efficiency of inserting a 150 bp DNA fragment at the target site was 63.0%, and the efficiency of inserting a 250 bp DNA fragment was 28.4%. It even allowed the insertion of a DNA fragment up to 1 kb, but the insertion efficiency was low when the inserted DNA fragment was larger than 400 bp. The study also confirmed that the PE system could effectively insert the target sequence at multiple genomic sites in multiple cell lines and non-dividing cells, which undoubtedly expanded the editing scope of the genome sequence by using the PE system, and made the targeted insertion of a large DNA fragment possible without providing the exogenous donor DNA. The GRAND editing lacked the step of using genomic DNA-RT product complementarities to solve the 3′flap. Therefore, it may have a low off-target effect. Based on the similar rule, the homologous 3′extension mediated prime editor system (HOPE) was developed (Zhuang et al., 2022). In this system, two pegRNAs, which contained the homologous 3′terminals and was used for targeting DNA double strands, were designed. Guided by the pegRNAs consisting of sense pegRNA and antisense-pegRNA, both strands of the target loci were edited simultaneously. HOPE provides a new choice of guided editing with a high balance between efficiency and accuracy. The potential off-target sites FANCF and HEK site3 of two representative genomic loci were analyzed to study off-target effects of HOPE, it found that HOPE induced extremely low substitution or indel ratios around predicted off-target sites.

Locating nick sgRNA near the pegRNA template sequence is helpful for targeted deleting the large fragments. Based on this rule, a two-way primer editing (Bi-PE) system was developed (Tao et al., 2022b). The system consisted of two engineered pegRNAs. One pegRNA carried the spacer and template sequences to guide the generation of single strand DNA breaks and subsequent extensions, and the other was used for creating gaps in the complementary strand. The Bi-PE system increased the editing efficiency by 16 times and the accuracy of editing products by 60 times. In addition, the system could effectively delete the large DNA fragments containing hundreds to thousands of base pairs. At the same time, this system could effectively introduce the small fragments of 10–100 bp in the deletion sites.

When the editing sequences contain more than 100 base pairs, the original PE system is inefficient. To solve this problem, the twin prime editing (twinPE) system was constructed (Anzalone et al., 2022). The system used a prime editor protein and two pegRNAs. Each of the two pegRNAs guided the editor protein to make a single strand gap at different target sites of the genome, avoiding the double strand break that generally produced unnecessary by-products. Then, the system synthesized two new complementary DNA strands, containing the required sequence between the two gaps. The length of genes that could be edited by twinPE expanded to thousands of base pairs which were equivalent to the length of most entire genes. When the twinPE system was combined with the site-specific recombinase, an inversion of nearly 40 kb DNA fragment which was related to Hunter syndrome in human cells was successfully edited. By amplifying and sequencing four characterized off-target loci in one of the *HEK3* spacer sequences, low off-target editing of PE was observed, demonstrating the potential application of twinPE in treatment of genetic diseases.

#### 3.1.2 Optimization of the template length for PBS and RTT

Since the PBS sequence and the reverse transcription template (RTT) in the PE system are important for the initial reverse transcription process, the melting temperature (Tm) becomes the key point for the stability of DNA, RNA, and DNA/RNA double stranded hybrid (Kaback et al., 1979). Therefore, the PBS sequence and its Tm value are inferred as the important factors affecting the editing efficiency of the PE system. Therefore, it is inferred that Tm of the PBS sequence is closely related to the editing efficiency of PPE, and may be one of the main factors affecting the design of pegRNAs. As shown in another study, the length of PBS (6–16 nt), the length of RT template (7–23 nt) and the position of nicking sgRNA in pegRNA had a strong impact on the editing efficiency (Lin et al., 2020; Lin et al., 2021). The ratio of targeted editing to off-target editing did not change when using the different PBS lengths or the different positions in nicking sgRNA, but it was significantly affected by the length of RTT. These results provide an important reference for the design of pegRNA in the PE system.

#### 3.1.3 Enhancing the stability of pegRNA

Expression and stability of pegRNA are the most important factors affecting the editing efficiency of the PE system. To improve the editing efficiency of the PE system, the pegRNA skeleton was optimized through deletion, replacement and other strategies of reducing its cyclization (Liu et al., 2021b). In this study, two aspects in the pegRNA skeleton were considered. First, the nicking sgRNA and pegRNA were fused to make them co-express in one transcriptional unit. In order to release the nicking sgRNA from the transcript, the effecter protein was fused with the Csy4 RNase (Csy4-T2A) which could recognize and cut the 3′end of the Csy4 recognition site. Second, the fourth base of the serial conserved uracil bases in pegRNA was mutated into cytosine to eliminate a possible termination signal in transcription, thus increasing the expression of pegRNA and the editing efficiency of the PE system. The resulting PE system was called the enhanced PE editing system (ePE). Compared with the standard PE system, the editing efficiency of ePE in the point mutation was increased by 1.9 times on average in the HeLa and N2a cells. However, ePE caused indel by-products, and the addition of Csy4 in the system hindered the delivery of the ePE system. Therefore, the ePE system should be further optimized.

It was found that the degradation of the 3′terminal extension region of pegRNA containing RTT and PBS reduced the activity of the PE system and hindered the editing efficiency (Nelson et al., 2022). Therefore, the engineered pegRNAs (epegRNAs) were constructed by integrating the RNA motifs with the specific structures into the 3′end of pegRNAs to enhance their stability and prevent degradation of pegRNAs from their 3′ends. The editing efficiency of optimized PE system was increased by 3–4 times in the HeLa cells, the U2OS cells, the K562 cells and the primary human fibroblasts without increasing the off-target editing activity. By measuring the extent of indel generation and nucleotide changes at the top four off-target sites, epegRNA and pegRNA showed similar off-target editing levels.

The Xrn1 resistant RNA (xrRNA) is a group of conserved structures found in flavivirus, which can enhance the anti-degradation ability of pegRNA. An upgraded PE system (xrPE) was developed through adding an xrRNA in the 3′extension region of pegRNAs (Zhang et al., 2022). In a given cell type, the average enhancement in base transformation, small deletion and small insertion was 3.1, 4.5, and 2.5 times, respectively. In addition, xrPE showed comparable edits:index ratios, and minimum deviation target edits similar to the specification of PE3. It is worth noting that the editing performance between xrPE and the currently developed epegRNA system is roughly the same.

#### 3.1.4 Optimization by improving pegRNA structure

Due to gRNA misfolding, some DNA sequences are resistant to CRISPR-Cas9 cleavage. To solve this problem, “locked” hairpin tracrRNA was designed which prevented misfolding of gRNA by introduction of highly stable hairpins (Riesenberg et al., 2022). In the PE system, this strategy was also valuable (Li et al., 2022). The apegRNA was developed by improving the pegRNA secondary structure and the spegRNA was developed by introducing same-sense mutations (SSM) at proper positions. With the apegRNA and the spegRNA, the editing efficiency of modified PE system was increased by 2.77 and 353 fold, respectively. The frequency of indel was also increased, but the frequency of unexpected indel, the part of incomplete products and by-products were not significantly affected. In addition to applying apegRNA and spegRNA separately, the strategy could be combined with other strategies to further improve the editing efficiency of PE.

#### 3.1.5 Optimization of pegRNA through software tools

The design of pegRNA in PE is more complex than CRISPR-Cas or BE, some software tools are available to solve this problem. PrimeDesign, a comprehensive and user-friendly software tool, was used to design pegRNA/ngRNA in installing a variety of human pathogenic variants (Hsu et al., 2021). The web tool called pegFinder could incorporate on-target and off-target scoring predictions of sgRNA, which has been used to design pegRNA for increasing editing efficiency (Chow et al., 2021). The computational models (DeepPE, PE_type and PE_position) could predict the efficiency of pegRNA with different lengths of PBS and RTT for edits of various types and positions in human cells (Kim et al., 2021). The potential off-target activities are crucial shortcomings in the PE system. Maximizing on-target activity and minimizing off-target effects could achieve more effective and efficient gene screening and genome engineering by combining large-scale empirical data, improving computational design rules, and creating an optimized sgRNA library. Cas-OFFinder could search for potential off-target sites in a given genome, which is not limited by the number of mismatches and allows variations in PAM sequences (Bae et al., 2014). The web tool CRISPOR is also helpful to improve editing efficiency by predicting off-targets and reduce the time spent on screening for off-targets (Haeussler et al., 2016).

Editing efficiency remains a significant challenge for PE in future. Engineering pegRNA is still the main method to improve the PE editing efficiency. The computational tools have the potential to simplify the successful use of prime editing and broaden its applicability. A more in-depth study of the mechanism of PE and the development of web software will also help provide new ideas for improving editing efficiency.

### 3.2 Optimization of PE based on effecter

Since the working mode and the activity of effecter determine the genome targeted editing efficiency and accuracy of the PE system, optimization of effecter becomes an important strategy for improving the PE system.

#### 3.2.1 Expand the editing scope of PE through constructing the effecter variants

The genome editing technology based on the CRISPR-Cas9 system is largely limited by the Cas9 recognition sites. For example, SpCas9 from *Streptococcus pyogenes* only recognizes the PAM sites with NGG sequence. It has been reported that the PAM sequence recognized by SpCas9 could be expanded by optimizing the Cas9 protein (Hu et al., 2018). Based on the structural analysis and optimization results of the Cas9 protein, various types of the PE2 variants (PE2-VQR, PE2-VRQR, PE2-VRER, PE2-NG, PE2-SpG, and PE2-SpRY) which could recognize the different PAM sequences were constructed, and activity of these PE2 variants was compared in the HEK293T cells (Kweon et al., 2021). Comparing other PE2 variants, these PE2-SpG variants were suitable for targeting the PAM sites with NGH sequence. The prime editing induced by PE2 SpRY variant had no PAM limitation, although the activity was reduced. Using the PE2-SpRY variant, the BRAF/V600E mutation with clinical significance was successfully introduced and the editing efficiency was more than 10%. These results indicated that the PE2-SpRY variant could provide more PE editing sites for the treatment of human genetic disease, improve the editing efficiency and avoid the unnecessary mutations. In order to apply PE to various biological systems, the basic limitations generated by CRISPR module must be overcome. To fulfill this purpose, a new system was developed by connecting the reverse transcriptase to the *Francisella novicida* Cas9 (FnCas9) (Oh et al., 2022). Comparing SpCas9, FnCas9 showed different characteristics on the non-targeted strand of the protospacer. Its advantage was to expand the recognized region for prime editing. This system confirmed that the editable region of primary editing could be extended by the different scoring characteristics of Cas orthologs and the construction of PAM recognition domain in Cas9 scoring.

#### 3.2.2 Optimization of the reverse transcriptase domain of effecter

By optimizing the reverse transcriptase domain of effecter, the engineered plant prime editor (ePPE) was constructed and applied (Zong et al., 2022). It was found that deleting the ribonuclease H (RNase H) domain in M-MLV-RT and adding the viral nucleocapsid protein (NC) could significantly improve the editing efficiency for multiple targets in rice and wheat, thus increasing the flexibility and applicability of PE. Compared with the original PPE system, the editing efficiency of ePPE in base substitution, deletion and insertion at different endogenous sites was increased by an average of 5.8 times, while no significant increase of by-products was observed. To study the effect of ePPE on off-target prime editing, the tolerance of ePPE to the mismatch in pegRNA was tested. It was found that both ePPE and PPE showed very low off-target efficiency at all examined sites, except for OsCDC48-T1. Using the ePPE system, the rice plants which were resistant to sulfonylurea and imidazolinone herbicides were successfully obtained, and the editing efficiency reached up to 11.3%. Besides optimizing the reverse transcription domain of M-MLV-RT, selecting the reverse transcriptase from different sources was considered as another strategy to optimize the effecter protein (Lin et al., 2020). Hopefully, more PE systems will be constructed with different reverse transcriptases in the future.

#### 3.2.3 Optimization of effecter by fusing RT and WT-Cas9

By fusing the reverse transcriptase (RT) and the wild-type nuclease Cas9, the WT-PE system was designed (Tao et al., 2022a). This system introduced DSBs and a single 3′extended flap into the target site. Combined with pegRNAs, this system could generate bi-directional primer editing. WT-PE realized the efficient and multifunctional large-scale genome editing, including the large-scale deletion of up to 16.8 Mbp and chromosome translocation. Other applications of WT-PE in chromosome engineering were also reported, including the inversion of inner chromosome fragments and the production of circular chromosomes or outer circular DNA.

#### 3.2.4 Optimization of effecter by separating RT and Cas9

The effecter of conventional PE system usually has a linker between RT and Cas9. However, the recent study found that the split nSpCas9 and MMLV-RT proteins in human cells showed the same efficiency as the intact PE2 system in which MMLV-RT could act in trans from another PE2 molecule and not tethered to the targeted site (Grunewald et al., 2022). The greatest advantage of this strategy is that it can be tested faster without optimizing the length or relative position of the linker with the fusion protein. In addition, off-target effect in the split PE2 system is similar to that in the standard PE2 system. The split PE2 system can also facilitate the delivery of PE components, and accelerate the further improvement of the PE system.

#### 3.2.5 Optimization of PE through inhibiting the DNA mismatch repair

Although the PE system has been widely used in different species, there is a lack of in-depth understanding of the mechanism that determines the editing efficiency of PE in cells. 476 genes involved in the process of DNA repair were studied by using the CRISPR interference technology (CRISPRi), and it was found that the DNA mismatch (MM) strongly inhibited the editing efficiency of the PE system and stimulated the occurrence of indels (Chen et al., 2021). Based on these findings, the editing efficiency of PE system was improved by transiently expressing the gene encoding the DNA mismatch inhibitory protein (MLH1dn). The improved system was called PE4 (PE2 + MLH1dn) and PE5 (PE3 + MLH1dn), respectively. The editing efficiency of PE4 and PE5 systems was 7.7 times and 2.0 times higher than that of PE2 and PE3 respectively, and the ratio of correct editing to off-targeted indels increased by 3.4 times. In addition, it was found that the DNA mismatch was eliminated by adding the silent mutations to the targeted sequence, which significantly improved the efficiency of targeted editing. A more efficient PE editing system (PEmax) was further constructed by changing RT codon, mutating SpCas9 and optimizing the NLS sequence. To sum up, the editing efficiency of PE system is greatly improved through using MLH1dn, PEmax and the optimized epegRNAs.

### 3.3 Collaborative optimization of PE

Combining the strategies of optimizing pegRNA and effecter, a variety of collaborative optimization strategies have emerged recently. As mentioned above, the PEDAR technology was constructed by combining the wild-type Cas9 with the reverse transcriptase, and applying a pair of pegRNAs (pegF and pegR) (Jiang et al., 2022a). Alteration of the linker between RT and nCas9 could be an effective approach to increase the efficiency of PE (Velimirovic et al., 2022). Through the Peptide Self-Editing sequencing assay (PepSEq), 105 candidates were identified as the prime editing enhancers in four human and mouse cell lines. Further studies showed that the linker IN-PE2 enhance the efficiency of PE probably through increasing the translation of the effecter encoding gene. Fusion of the reverse transcriptase in the N-terminal of nCas9 was better than the fusion in the C-terminal of nCas9 and introduction of multiple nucleotide substitutions in RTT significantly improved the editing efficiency of PE (Xu et al., 2022). Through the collaborative use of these two methods, PE was successfully applied in the rice transgenic plants, maize protoplasts and human cells. The editing efficiency reached to 24.3%, 6.2% and 12.5% respectively, which was 2–3 times higher than that of the original PE3 system.

Due to the inevitable base pairing between PBS and the original spacer sequence, and the potential interaction between RTT and pegRNA scaffold, RNA misfolding easily occurs. Moreover, the 3′extension end of pegRNA is exposed and easy to be degraded by nuclease, which is not conducive to the stability of pegRNA. In order to reduce the RNA misfolding and instability of pegRNA, a split PE system (SPE) was developed (Liu et al., 2022). They split the pegRNAs into sgRNA and the circular RNA RT template (petRNA) to increase flexibility and stability of pegRNAs, and replaced M-MLV-RT with two compact codon optimized bacterial RT (*Eubacterium rectale* maturase RT and GsI-IIC RT). In SPE, a dual AAV vector strategy was used. *nCas9* was expressed in one AAV vector, and RT, pegRNA and sgRNA were expressed in the second AAV vector. Comparing PE, SPE is more conducive to delivery *in vivo*, and its editing efficiency is similar to that of PE3. Meanwhile, it does not increase production of the off-target indels.

### 3.4 Comprehensive optimization strategies

Because each strategy of optimizing pegRNA, optimizing the effecter proteins or inhibiting the DNA mismatch repair has certain limitations. Combining strategies (such as ePE3max and ePE5max) were constructed and applied in rice (Jiang et al., 2022b). The ePE3max system consists of a PEmax protein, an epegRNA with evopreQ1 and a nicking sgRNA. Based on ePE3max, the ePE5max system was constructed by introducing a dominant negative OsMLH1 variant which could inhibit MMR. To study non-transgenic glyphosate resistant rice breeding, ePE3max and ePE5max were applied in rice EPSPS to generate the suitable TAP-IVS mutations. The results demonstrated that the prime editing efficiency in rice was greatly increased by improving PEmax structure and epegRNA. In addition, the by-products derived from pegRNA scaffold could be eliminated by using the termination rule of designed pegRNA. The optimization of the PE system in future is certainly comprehensive, the study on the structure of PE - pegRNA - DNA target complex may provides information for designing more active prime editor architecture.

## 4 Summary and prospect

The PE system based on CRISPR-Cas9 can be used for deletion, replacement and insertion of the targets in genome, and it shows extraordinary versatility, product purity and target specificity. Since this technology emerged 3 years ago, it has shown great potential in engineering the cell lines or strains with new functions or phenotypes and creating new platforms which are used for producing enzymes, chemicals and so on. As the most rapidly developing research field in life science, the development and application of the genome editing technologies, including PE, has brought the genetic engineering of organisms into an unprecedented depth and breadth. Genome editing technology based on CRISPR-Cas9 has become a powerful and widely used genetic tool. Recently, PE has added more powerful functions to genome editing. As mentioned in this review, these outstanding studies highlight characteristics of the PE system, such as high efficiency, less by-products and wide editing scope (Xu et al., 2020; Caso and Davies, 2022). It is believed that with the further optimization and updating of this technology, PE will have a far-reaching impact on basic research, precision molecular breeding and disease treatments.

Comparing the BE system, the PE system is more efficient and has less by-products. However, in the classic base editing window, the BE system has higher editing efficiency and less risk of producing off-targeted indels. If the designed nucleotide location is poor, the PE system is more efficient because it is less dependent on the PAM sites (Anzalone et al., 2019; Marzec et al., 2020). Although the PE system has made some breakthroughs, there are still some deficiencies. First, the editing efficiency of PE needs to be improved further. Through constantly optimizing, the efficiency of PE system has been greatly increased compared with that of the original PE system. However, comparing the CRISPR-Cas system, the editing efficiency of PE is still very low. Meanwhile, it was reported that the editing efficiency of PE system was various in different species (Lu et al., 2022), which could be related to the expression level of PE effecter protein and pegRNA in different species. Second, the ratio of off-target editing is needed to be reduced and the mechanism of off-target editing is needed to be clarified. As a newly developed editing system, the editing mechanism of PE system is poorly understood due to lacking systematic and in-depth studies. This not only limits the further improvement of editing efficiency, but also hinders the rational reduction of off-target editing. Fortunately, several studies related to the editing mechanism of PE have been published recently, such as the relationship among DNA mismatch repair pathway (MMR), editing efficiency and the generation of indel by-products (Jiang et al., 2022b). Third, the universality of PE system needs to be improved for its application in a wider range of cell types and organisms. Although PE has been widely applied in animals, plants and microorganism, but its application is mainly limited to the important animals, plants and the model microorganism *E. coli*. It is expected that PE can be widely used in more species, especially in the microorganisms with relatively simple genetic background, which will in turn promote the further development of the PE technology. Therefore, the PE system still needs iterative updating to solve the problems of its specificity, effectiveness and safety.

In summary, improving the editing efficiency of the PE system is urgently needed recently. The PE system consists of the effecter protein and pegRNA (Anzalone et al., 2019). Therefore, the editing efficiency of PE could be optimized first from these two aspects, including optimizing pegRNA and the PE effecter protein respectively. In addition, the strategy of optimizing pegRNA and effecter synergistically has been applied recently. Although extensive efforts have been made to increase the editing efficiency and expand the editing scope, PE is still a new tool at the early stage of development and needs further improvement. As the most concerned issue of gene editing, safety needs to be paid more attention. The off-target effect is a common problem of PE. Although many studies have shown that the off-target of PE is relatively low, an imperceptible deviation from the target may also lead to unpredictable or even fatal consequences. Therefore, it is more necessary to carry out comprehensive study of the off-target effect in different organisms, different development stages and different physiological conditions. In future, PE should be developed into a high-throughput platform to achieve more applications. It is believed that with the continuous improvement of genome editing technology, PE technology will be used in animal, plant and microbial breeding and improvement, disease treatment and other fields of landmark breakthroughs. In addition, the combination of mutual strategies with other internal diplomacy will also bring revolutionary changes to the field of disease treatment.
